# Correspondence to paper by Malik A: “Meckel’s Diverticulum-Revisited”

**DOI:** 10.4103/1319-3767.65180

**Published:** 2010-07

**Authors:** Yavuz Beyazit, Murat Kekilli, Mevlut Kurt

**Affiliations:** Department of Gastroenterology, Turkiye Yuksek Ihtisas Teaching and Research Hospital, Ankara, Turkey. E-mail: yavuzbeyaz@yahoo.com

Sir,

We read with great interest the review of Malik *et al*.[1] regarding the current understanding of Meckel’s diverticulum. The article gives an excellent overview of the field and the direction of current research. Because the scientific field is undergoing rapid development, making possible the use of tools and techniques that are new and entirely different, we would like to point out two additional techniques for diagnosing MD, namely, video capsule endoscopy (VCE) and double balloon enteroscopy (DBE). Both of these are novel methods of enteroscopy with a high diagnostic value.

VCE is a new method used for the diagnosis of diseases of the small intestine, with satisfying results. When all invasive/noninvasive attempts fail to detect an intestinal pathology, VCE could help to achieve a diagnosis. It has been introduced to fill the gap between examinations of the upper and lower gastrointestinal tract, mainly to examine the small bowel for sources of obscure bleeding in addition to various other indications, including MD.[2] The procedure is thought to be harmless, minimally invasive, highly sensitive and specific for digestive tract disorders. It requires no sedation or radiation, and can even be performed in outpatients. Although safety and tolerability is well known in adults, recent studies show successful results in pediatric patients.[3] The first report of detection of a MD using a VCE was reported by Mylonaki *et al*.[4] Since that publication, a large number of reports are available in the literature documenting a high success rate for diagnosis of MD using VCE.

DBE is an exciting new endoscopic technique that was developed to visualize the entire small intestine and has become available for clinical practice. It was first described by Yamamato and colleagues in 2001.[5] The system consists of an enteroscope, an overtube, and a balloon pump controller. The enteroscope has a working length of 200 cm, an outer diameter of 8.5 mm with a 140 cm overtube. DBE has two balloons, one connected to the tip of the endoscope and another at the distal end of the overtube that facilitates the progression of the endoscope through the small bowel. Loops can be easily reduced by gentle withdrawal of the endoscope while the balloons are inflated. With this technique, the endoscope can be advanced by pushing it through the overtube, allowing endoscopic visualization of the entire small bowel, as well as biopsies and therapeutic intervention.[6] Despite the technical challenges of the procedure and the resources required, it is clearly the preferred technique for treating bleeding lesions in the intestine and obtaining tissue for definitive diagnosis. Compared with VCE, it has numerous advantages. It is much more easily adjusted since the movement of the scope can be handled according to the viewing angle. It can afford high-quality pictures and allow biopsy if needed.[7] Obscure bleeding due to MD can be successfully diagnosed with this technique, which has also been reported in several papers.[8]

In conclusion, both these techniques allowed physicians to evaluate intestinal areas that were until recently difficult with conventional methods. Although these two tests may be capable of complementing each other, what could be the thought that they are in direct competition. The significance of these methods could further be improved by expanding the availability of adapted accessories.
